# Dupilumab as an effective therapy for eosinophilic esophagitis in pediatric patients weighing less than 15 kilograms

**DOI:** 10.1002/jpr3.70129

**Published:** 2025-12-12

**Authors:** Sindhura Kasturi, Tanaz Danialifar, Thirumazhisai Gunasekaran, Nick Shillingford, Paul Zamiara, Vrinda Bhardwaj, Melinda Braskett

**Affiliations:** ^1^ Department of Pediatric Gastroenterology, Hepatology and Nutrition Children's Hospital Los Angeles Los Angeles California USA; ^2^ Department of Pathology Children's Hospital Los Angeles Los Angeles California USA; ^3^ Department of Pathology and Laboratory Medicine The Hospital for Sick Children Toronto Ontario Canada; ^4^ University of Toronto Toronto Ontario Canada; ^5^ Department of Pediatric Allergy and Immunology Children's Hospital Los Angeles Los Angeles California USA

**Keywords:** allergy, EoE, monoclonal antibody

## Abstract

**Objectives:**

Eosinophilic esophagitis (EoE) is a type 2 cytokine‐mediated chronic inflammatory condition leading to esophageal dysfunction. Dupilumab blocks IL‐4 and IL‐13 signaling, which are key inflammatory mediators in EoE and other allergic disorders. In the United States, the Food and Drug Administration (FDA) has approved the use of dupilumab in children and adults with doses that vary by age, weight and indication. Dupilumab is currently approved for EoE in children above 1 year of age and weight greater than 15 kg. There is an unmet need to extend the use of dupilumab for children with EoE below this weight threshold and there is a paucity of data. We seek to describe the safety and efficacy of dupilumab in treating EoE at doses approved for eczema in children weighing less than 15 kg.

**Methods:**

The retrospective chart review was approved by the Institutional Review Board (IRB) at Children's Hospital Los Angeles. A search for patients with a diagnosis of EoE treated with dupilumab for any indication between 2019 and 2024 identified four patients weighing less than 15 kg.

**Results:**

This report highlights a single‐center experience with dupilumab in four patients with EoE weighing less than 15 k. All patients had clinical improvement. Three patients had follow‐up endoscopy demonstrating endoscopic and histologic improvement. No significant adverse effects were noted.

**Conclusions:**

In our limited experience, dupilumab was safe and effective in four EoE patients weighing less than 15 kg using the FDA‐approved dose for atopic dermatitis. Prospective trials are needed to optimize dosing and provide comprehensive safety and efficacy data.

## Abstract (highlights)

**What is Known:**

• Dupilumab is approved for treatment of eosinophilic esophagitis (EoE) in pediatric patients greater than 1 year of age and greater than or equal to 15 kg.
• Dupilumab is approved for treatment of atopic dermatitis in patients at least 6 months of age and at least 5 kg.
• Multiple studies have demonstrated that dupilumab dosing for atopic dermatitis can be effective in treating EoE patients greater than 15 kg.

**What is New:**

• In a small cohort, dupilumab was found to be safe and effective for the treatment of EoE in patients less than 15 kg at atopic dermatitis approved doses.

## 1 INTRODUCTION

Eosinophilic esophagitis (EoE) is characterized by esophageal inflammation, with greater than or equal to 15 eosinophils per high power field (eos/hpf) and symptoms of esophageal dysfunction. Food elimination diets, proton pump inhibitors (PPI), and swallowed topical corticosteroids (STS) are established therapies for the management of EoE. In addition to these, dupilumab, a humanized monoclonal antibody has emerged as a safe and effective treatment for EoE. Dupilumab inhibits IL‐4 and IL‐13 signaling by binding at their cell surface receptor. This reduces cytokine induced allergic inflammatory responses that are key drivers in EoE and other type 2 inflammatory disorders.^1^ Dupilumab is approved by the Food and Drug Administration (FDA) for the treatment of atopic dermatitis, asthma, chronic rhinosinusitis with nasal polyps, and most recently, EoE. FDA approved doses vary by age, weight and indication for younger patients. The cumulative monthly dose of dupilumab for EoE is higher for the same age and weight ranges compared to other indications. Dupilumab is approved for the treatment of EoE in children greater than 1 year old with weight greater than or equal to 15 kg based on data from a prospective randomized placebo control study involving 61 patients aged 1–11 years weighing ≥ 15 kg.^2^ Both prospective and retrospective data demonstrate efficacy of dupilumab in treating EoE at doses for other indications in children weighing greater than 15 kg.^2^, ^3^, ^4^, ^5^ At the time of this report, there is no published data regarding the use of dupilumab in patients with EoE weighing less than 15 kg.

## 2 METHODS

A search for patients with a diagnosis of EoE treated with dupilumab for any indication between 2019 and 2024 identified four patients weighing less than 15 kg out of 67 total patients. Clinical features, treatment and outcomes of these four pediatric patients are summarized below.

### 2.1 Ethics statement

The retrospective chart review was approved by the Institutional Review Board (IRB) at Children's Hospital Los Angeles. The parents of the subjects of the case series are aware of the intent to publish and have provided informed consent.

## 3 RESULTS

**Figure 1:**
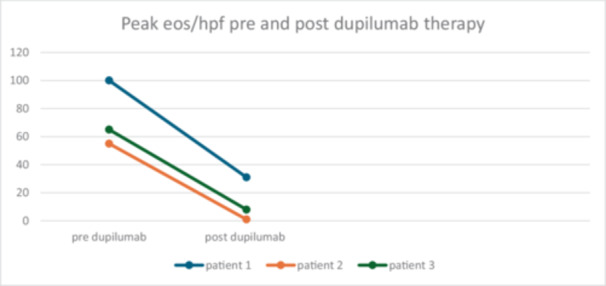
Change in peak eosinophils/high power field (eos/hpf) pre and post‐dupilumab therapy.

**Figure 2:**
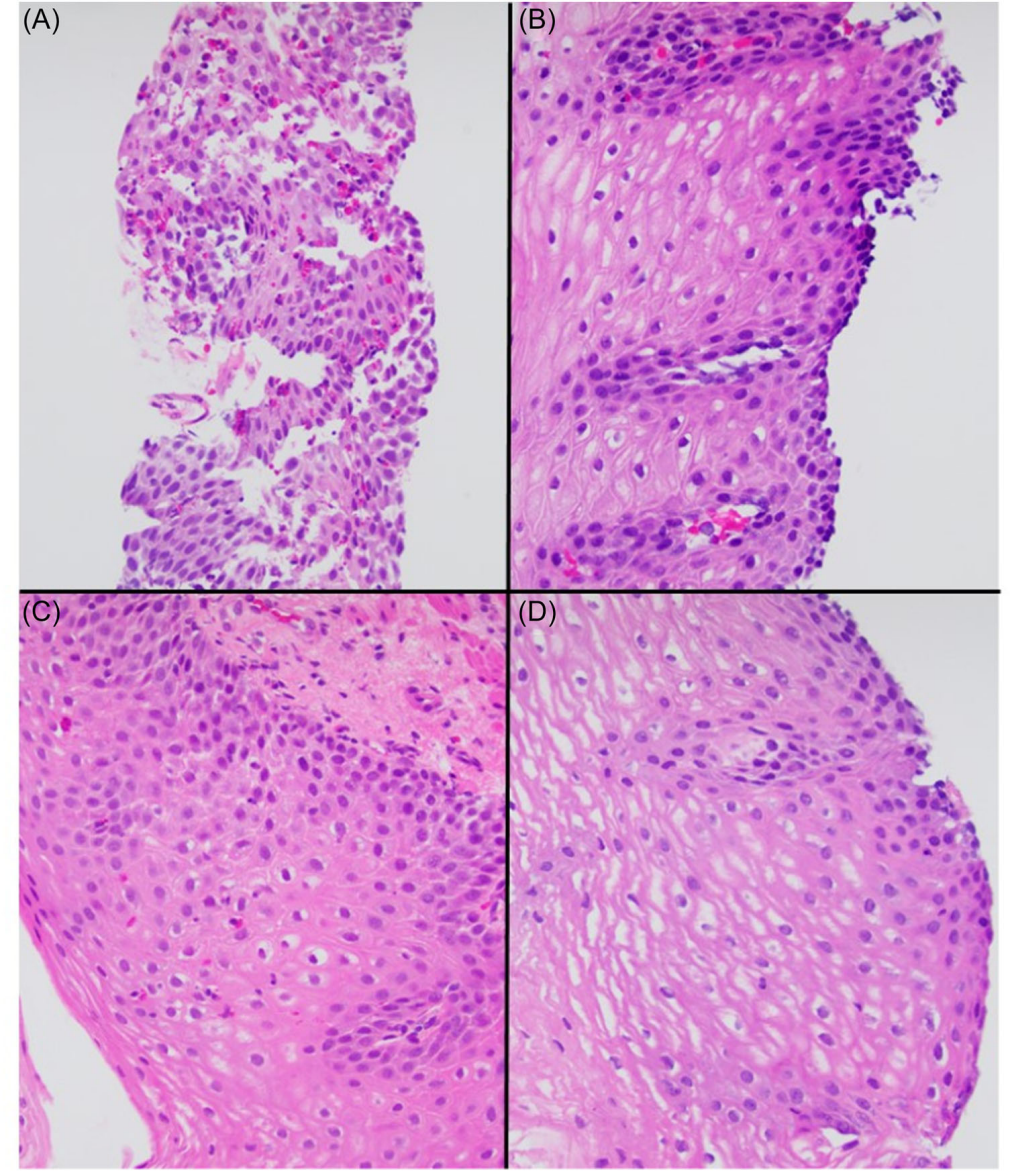
Patient 1 lower esophageal biopsies taken pre‐ (A & C) and post‐treatment (B & D) depict basal cell hyperplasia, spongiosis, and increased intraepithelial eosinophils including eosinophil degranulation in the pre‐treatment biopsies, with resolution or marked decrease in all features post‐treatment. Panels A & B are biopsies taken from the same patient, while C & D are from a different patient. H&E stained, at 20x (Panel A) or 40x (Panels B–D) magnification.

This case series describes four patients diagnosed with EoE at a very young age weighing less than 15 kg treated successfully with dupilumab 200 mg every 4 weeks. The male to female ratio was 3:1. At the time of initiation of dupilumab, the age range was 21–30 months old, and the weight range was 10.8–11.7 kg (11.1 kg). Duration of dupilumab therapy at the time of this report ranged from 13 months to 27 months. (Table 1) Three patients had repeat endoscopy which demonstrated significant histological improvement (Table 1, Figures 1 and 2). All patients had significant clinical improvement and expanded their diet successfully. When present, co‐morbid eczema also responded favorably to dupilumab therapy. No adverse safety effects related to dupilumab administration were demonstrated. One family struggled with anxiety related to home subcutaneous injections.

**Table 1:** Patient demographics and clinical characteristics.

	Patient 1	Patient 2	Patient 3	Patient 4
Age^a^	21 months	34 months	30 months	29 months
Sex	F	M	M	M
Weight^a^	10.8 kg	11.7 kg	11.3 kg	10.9 kg
Dupilumab indication	Atopic dermatitis	EoE‐impairment in growth with swallowed steroids	Atopic dermatitis	Atopic dermatitis
EoE symptoms	Vomiting, poor weight gain	Vomiting, oral aversion, poor weight gain	Vomiting, oral aversion, poor weight gain	Vomiting, cough, oral aversion, poor weight gain
Treatment before initiating dupilumab	Diet elimination, STS	Diet elimination, STS	Diet elimination, high dose PPI	Diet elimination, STS high dose PPI
Initial EREFS	4	0	0	3
Initial histology^b^	Proximal: >100 Mid: >100 Distal: 47	Proximal: 2 Mid: 55 Distal: 50	Proximal: n/a Mid: 65 Distal: 63	Proximal: 45 Mid: 50 Distal: 55
Time to endoscopic re‐assessment	6 months	8 months	4 months	n/a
Change in symptoms	Resolution of vomiting	Resolution of vomiting	Improved oral intake, resolution of vomiting, improved growth	Improved oral intake, resolution of vomiting and cough, improved growth
Expanded diet	Yes	Yes	Yes	Yes
Follow‐up EREFS	0	0	0	n/a
Follow‐up histology^b^	Proximal: 1 Mid: 31 Distal: 5	Proximal: 0–1 Mid: 0–1 Distal: 0–1	Proximal: n/a Mid: 8 Distal: 1	n/a
Duration of dupilumab therapy^c^	13 months	17 months	27 months	15 months

Abbreviations: EREFS, Eosinophilic Esophagitis Endoscopic Reference Score; PPI, proton pump inhibitor; STS, swallowed topical steroids.

a At time of dupilumab initiation.

b Eosinophils/high power field.

c By the time of submission of manuscript.

### 3.1 Patient 1

A 17‐month‐old female with multiple food allergies and eczema presented to our institution with oral aversion, poor weight gain, and vomiting, starting at 4 months of age. Vomiting and food aversion continued despite an extensive food elimination diet. Atopic dermatitis was poorly controlled even with topical steroids and topical tacrolimus therapy. Initial esophagogastroduodenoscopy (EGD) while patient was on extensive elimination diet demonstrated edema, exudates, and linear furrows without strictures (EREFS score 4). Histology revealed more than 100 eos/hpf in the proximal and mid esophagus and 47 eos/hpf distally, with eosinophilic micro‐abscesses (Table 1). Biopsies of the stomach and duodenum were normal. Treatment was initiated with dupilumab 200 mg every 4 weeks as well as swallowed budesonide slurry 0.5 mg twice daily as a bridge until dupilumab reached steady state level.

After 3 months of dual therapy, emesis and feeding behaviors improved and budesonide was discontinued as parents did not wish to prolong treatment with corticosteroids. She was maintained on dupilumab. Eczema was well controlled, and she tolerated reintroduction of fish and legumes in addition to multiple lower‐risk foods that previously triggered emesis. Six months after initiating dupilumab (3 months after discontinuing budesonide) follow up EGD was visually normal with EREFS score of 0, and histology demonstrated dramatic improvement with 1 eos/hpf proximally, 31 eos/hpf in the mid esophagus, and 5 eos/hpf distally with resolution of the eosinophilic micro‐abscesses. Given substantial clinical improvement and ability to reintroduce multiple high risk and trigger foods, no additional therapy was added. Eczema remained well controlled. At the time of this report, she received over 13 months of dupilumab therapy without any adverse events. Repeat endoscopic evaluation was deferred due to family preference and sustained clinical improvement.

### 3.2 Patient 2

A 2‐year‐old male presented with cough, vomiting, oral aversion, and poor weight gain. EGD at presentation was grossly normal. Histology revealed 50 eos/hpf distally, 55 eos/hpf in the mid esophagus, and 2 eos/hpf proximally with fibrosis of lamina propria. Biopsy samples of the stomach and duodenum were normal. After 3 months of therapy with budesonide slurry 0.5 mg twice daily and a six‐food elimination diet, oral aversion and vomiting had resolved. EGD was grossly normal and esophageal biopsies demonstrated less than 6 eos/hpf in all areas. His diet was liberalized to include fish and nuts, and he continued four‐food elimination. Due to linear growth stagnation budesonide was discontinued. Despite symptomatic control with four‐food elimination diet, repeat EGD demonstrated recurrence of EoE with 45 eos/hpf in mid esophagus and 12 eos/hpf distally with eosinophilic micro‐abscesses. Therapy with dupilumab 200 mg every 4 weeks was initiated, as a compassionate use due to concerns of STS negatively impacting linear growth and food restrictions affecting oral intake. Dietary restriction was discontinued and after 8 months of dupilumab therapy repeat EGD was grossly normal with 0–1 eos/hpf in all three areas of the esophagus and without micro‐abscesses or fibrosis. At the time of this report, he received over 17 months of dupilumab therapy without any adverse events.

### 3.3 Patient 3

A 22‐month‐old male, with food allergies, poorly controlled eczema, and recent diagnosis of EoE presented to our institution for a higher level of care. Initial presenting symptoms were vomiting, oral aversion and poor weight gain. EGD at diagnosis was normal (EREFS 0) and histology revealed 65 eos/hpf in the distal and 63 eos/hpf in the mid esophagus. Stomach and duodenal biopsies were normal. Vomiting and oral aversion failed to respond to extensive elimination diet (dairy, soy, eggs, nuts, fish) so high dose PPI therapy was added. Repeat EGD was grossly normal with EREFS 0, and biopsies showed 13 eos/hpf distally. Despite histologic improvement, he continued to demonstrate food aversion and vomiting. Parents did not wish to pursue STS therapy or further restrict his diet. Dupilumab was initiated for management of poorly controlled atopic dermatitis at a dose of 200 mg every 4 weeks. After 4 months of therapy with dupilumab, the patient had improvement in oral aversion. Eliminated foods were reintroduced except for eggs due to risk of immediate hypersensitivity. PPI was discontinued. Repeat EGD on dupilumab was normal (EREFS 0) and biopsies showed 8 eos/hpf in mid esophagus and 1 eos/hpf in distal esophagus. Eczema was controlled. After over 27 months of therapy there have been no significant adverse effects. However, the patient developed anxiety related to injection administration that did not result in discontinuation of therapy.

### 3.4 Patient 4

A 2‐year‐old male with eczema, cough with feeding, vomiting and poor weight gain was diagnosed with EoE at another institution. EGD at diagnosis demonstrated EREFS score of 3 and biopsies showed 45 eos/hpf in the proximal esophagus, 50 eos/hpf in the mid esophagus and 55 eos/hpf distal esophagus with 100 eos/hpf in the stomach and 50 eos/hpf in the duodenum. He was initially treated with six‐food plus corn elimination diet and STS. After approximately 1 month of therapy with STS, he developed oral ulcers that were resolved with cessation of STS. At the time of presentation to our institution, he was maintained on the elimination diet and had ongoing growth failure and poorly controlled atopic dermatitis. Additionally, the family felt dietary therapy was not sustainable. No improvement in symptoms was noted after 6 weeks of high dose PPI therapy. He was started on dupilumab 200 mg monthly for management of eczema. He had clinical improvement with resolution of cough and vomiting, gained weight and tolerated expansion of the diet to include wheat, eggs, seafood, soy, and legumes. He has since transferred care and no additional endoscopy has been completed. At the time of this report no adverse events related to dupilumab were noted after 15 months of therapy.

## 4 DISCUSSION

This case series reflects real‐world experience in the management of EOE in very young children weighing less than 15 kg. Given the limitations in management and disease monitoring within this patient population, success was defined not only by endoscopic and histologic findings, but also ability to wean other therapies with reported side effects, improvement in clinical symptoms and feeding and ability to liberalize the diet without clinical or histologic worsening. In this case series, all patients had prior treatment with suboptimal responses to food elimination diets. PPI therapy was tried and failed in two children. STS therapy was associated with potential adverse effects in two patients (linear growth stagnation and oral ulcers) and the remaining two families were resistant to prolonged therapy with STS. Importantly, all families were interested in liberalizing the dietary restrictions. All patients weighed less than 12 kg and experienced poor weight gain, thus the weight threshold of 15 kg for the EoE indication was not attainable. Three patients had co‐morbid atopic dermatitis that served as the indication for dupilumab therapy, and one patient received authorization given steroid intolerance. One patient had eosinophilic gastroduodenitis in addition to EOE.

Management of EoE is challenging in the pediatric population. High‐dose PPI, swallowed topical steroids, and dietary therapy are established treatment options. Each treatment strategy has potential benefits and limitations that require careful consideration to optimize patient outcomes and facilitate family centered care.^6^, ^7^, ^8^, ^9^, ^10^, ^11^, ^12^, ^13^, ^14^, ^15^, ^16^, ^17^, ^18^, ^19^, ^20^, ^21^, ^22^, ^23^, ^24^, ^25^, ^26^ Response rates are difficult to compare as standards are evolving.^27^

Dupilumab was recently approved to treat EoE in children with efficacy rates comparable to that observed in adolescents and adults.^2^, ^3^ Injection site reaction (13%) was the most reported adverse event followed by Covid 19 (9%), diarrhea, pyrexia and fatigue (6% each) in the 1‐year clinical trial with EoE dosing for children ages 1–11 years.^2^

The cumulative monthly dose of dupilumab for EoE is higher than for other atopic conditions (Table 2). However, data from pivotal Phase 2 and Phase 3 randomized placebo controlled clinical trials determined that non‐EoE doses can be effective in treating EoE.^2^, ^28^ Additionally, two case series studying a total of 52 patients ages 6 and up demonstrated positive outcomes and clinical improvements in EOE while receiving dupilumab for other conditions.^4^, ^5^ These studies, like ours, demonstrated clinical as well as histological improvement in patients treated with non‐EoE dosing but lacked data on younger patients with lower weight ranges.

**Table 2:** Dupilumab pediatric dosage by indication for age and weight ranges.

Indication	Age range	Maintenance dose
Atopic dermatitis	> = 6 months	5 to <15 kg 200 mg every 28 days 15 to <30 kg 300 mg every 28 days 30 to <60 kg 200 mg every 14 days > = 60 kg 300 mg every 14 days
Asthma	6 to < = 12 years > = 12 years	15 to <30 kg 300 mg every 28 days > = 30 kg 200 mg every 14 days 200 mg or 300 mg every 14 days (both acceptable)
Chronic rhinosinusitis with nasal polyps	> = 12 years	300 mg every 14 days
Eosinophilic esophagitis	1 + years	15 to <30 kg 200 mg every 14 days 30 to <40 kg 300 mg every 14 days > = 40 kg 300 mg every 7days

This case series of four young patients with EoE weighing less than 15 kg treated with dupilumab at 200 mg monthly begins to address this unmet need for safety and efficacy data for this group of patients. Although dupilumab was generally well tolerated, we uncovered high levels of anxiety in at least one patient and his family regarding administration. Parental and patient anxiety related to home subcutaneous injections may prove to be a barrier to dupilumab therapy in younger patients. Despite this barrier, in our cohort, it did not limit continuation of therapy.

Limitations of this study include lack of placebo controlled prospective data, small sample size, lack of standardization in concomitant therapies, limited endoscopic follow‐up, and inclusion of one subject with extra‐esophageal involvement. We acknowledge that the lack of endoscopic follow up on 2/4 patients is a major limitation in assessing effectiveness of this therapy. Nonetheless this data is important to report given the clinical improvements noted and the fact that the inability to repeat endoscopy in all patients reflects real‐world experience, where decisions may need to be made on the basis of incomplete or imperfect data. Prospective clinical trials with well characterized cohorts are needed to assess the generalizability of these results for patients at this weight range. At this time treatment for children with EoE weighing less than 15 kg remains investigational.

## 5 CONCLUSION

In our limited experience, dupilumab was safe and effective in four EoE patients weighing less than 15 kg using the FDA‐approved dose for atopic dermatitis. These findings suggest that EoE patients weighing at least 5 kg but less than 15 kg, who are unresponsive, unable or unwilling to tolerate other therapies, may be treated with dupilumab 200 mg monthly. Prospective trials are needed to optimize dosing and provide comprehensive long‐term safety and efficacy data.

## CONFLICT OF INTEREST STATEMENT

The authors declare no conflicts of interest.
